# Apoptosis Induction by dsRNA-Dependent Protein Kinase R (PKR) in EPC Cells via Caspase 8 and 9 Pathways

**DOI:** 10.3390/v10100526

**Published:** 2018-09-27

**Authors:** Cheng Xu, Amr A. A. Gamil, Hetron Mweemba Munang’andu, Øystein Evensen

**Affiliations:** Faculty of Veterinary Medicine, Norwegian University of Life Sciences, PO Box 369, 0102 Oslo, Norway; cheng.xu@nmbu.no (C.X.); amr.gamil@nmbu.no (A.A.A.G.); hetroney.mweemba.munangandu@nmbu.no (H.M.M.)

**Keywords:** apoptosis, annexin-V, caspase 8 and 9, eIF2alpha, phosphorylation, PKR

## Abstract

dsRNA-dependent protein kinase R (PKR) is an interferon-inducible protein that mediates antiviral effects and induces apoptosis. We studied PKR-related apoptosis mechanisms by transfecting wild type pcDNA-carp-wtPKR, a catalytically inactive mutant pcDNA-mut-carpPKR, and empty plasmid in *Epithelioma papulosum cyprini* (EPC) cells, designated wtPKR, mutPKR, and pcDNA3.1, respectively. PKR was inefficiently expressed from wtPKR unlike mutPKR that produced high PKR levels detected by western blot. eIF2α phosphorylation increased in wtPKR-transfected cells, while for mutPKR, phosphorylation was not different from non-transfected controls. Flow-cytometry revealed high level of apoptosis in wtPKR transfected cells, corresponding with high cytopathic effect. mutPKR and pcDNA3.1 transfection gave significantly less apoptosis and were not different from each other. Caspase-8 and -9 were activated for wtPKR, suggesting death receptor-caspase-8 and mitochondrion-dependent caspase-9 activated pathways, similar to mammalian cells. These findings suggest that the induction of apoptosis via the caspase-8 and -9 pathways are conserved in vertebrate taxa and likely play a role in viral infections of lower vertebrates.

## 1. Introduction

Type I interferon (IFN) response is the major innate immune defense mechanism against viral infection. Production of type I IFN is stimulated by recognition of invading viruses through different host sensors [1]. Once secreted, type I IFN binds to its receptor on the cell surface in an autocrine or paracrine manner and triggers a signaling cascade through the Janus kinase/signal transducer and activator of transcription proteins (JAK/STAT) pathway [2,3]. Induction of different IFN-stimulated genes (ISGs) leads to the establishment of an antiviral state in host cells [4]. The dsRNA activated protein kinase R (PKR) is an ISG constitutively expressed in nearly all mammalian cells and is activated by the binding of dsRNA to dsRNA-binding motifs (dsRBMs) at its N-terminus [5,6,7]. Activated PKR undergoes dimerization, which subsequently promotes autophosphorylation to produce the active form [8]. The best known function of the activated PKR is the control of protein translation via phosphorylation of the alpha subunit of eukaryotic initiation factor 2 (eIF2α), whose main function is inhibition of protein synthesis in order to prevent viruses from producing new progeny [9]. eIF2α is the primary substrate of PKR and its phosphorylation correlates with the induction of programmed cell death that renders it to serve as a molecular determinant of apoptosis [10]. PKR has been identified, cloned, and characterized in several fish species such as olive flounder (*Paralichthys olivaceus*) [11], zebrafish (*Danio rerio*) [12], crucian carp (*Carassius carassius*) [13], and rock bream (*Oplegnathus fasciatus*) [14]. In addition, it has been shown to phosphorylate eIF2α and its antiviral properties have been demonstrated in flounder and carp [11,15]. As part of the antiviral responses, PKR mediates cellular apoptosis but can also induce apoptosis in the absence of a virus infection [5]. There are studies on the involvement of PKR as part of a viral infection in fish [16,17] but few addressing the induction of apoptosis as an integral part of the antiviral response. Hu et al. [17] allude to the cellular responses mediated by PKR and eIF2α phosphorylation but did not perform any analysis to confirm this. Induction of apoptosis in fish cells in the absence of viral infection remains unexplored.

There are two major apoptosis pathways: (i) the extrinsic pathway commonly referred to as the “death receptor-mediated pathway”; and (ii) the intrinsic mitochondrial pathway [18,19]. The extrinsic pathway uses transmembrane death receptors that are members of the tumor necrosis factor (TNF) genes superfamily, that share a similar-cysteine-rich extracellular domain called the “death domain” [20]. This domain transmits death signals from the cell surface to intracellular ligands to the apoptosis cellular machinery. The best characterized death receptor-mediated interaction is the FS7-associated cell surface antigen (Fas) receptor-ligand interaction, and engagement leads to binding of the adapter protein Fas-associated death domain (FADD) followed by formation of the death inducing complex (DISC) and auto-catalytic activation of caspase-8 [21]. The Intrinsic signaling pathway uses non-receptor mechanisms that activate the caspase dependent mitochondrial pathway where cytochrome c binds and activates the apoptosis protease activating factor (Apaf)-1 together with procaspase-9 to form a complex called “apoptosome” [22]. Involvement of procaspase-9 leads to activation of caspase-9 that initiates the apoptosis execution phase by activating the downstream caspase cascade [23,24]. Ultimately, the caspase-8 and -9 pathways converge on activating the terminator caspases, caspase-3, -6, and -7 that are executioners of apoptosis [25,26]. Caspase-8 and -9 genes have been reported in fish [27,28,29,30] but their involvement in induction of apoptosis has not been explored. On this basis, we were interested in studying the mediation of PKR in apoptosis in the absence of a virus infection and we used an approach of PKR overexpression, monitored phosphorylation of eIF2α and also included an assessment of involvement of caspase-8 and -9 in the process. We find that PKR overexpression in *Epithelioma papulosum cyprini* (EPC) cells induces apoptosis following eIF2α phosphorylation, and activation of caspase-8 and -9. These responses are ablated when transfecting with a PKR variant with a mutated, catalytically inactive domain.

## 2. Materials and Methods

### 2.1. Cell Culture and Virus

*Epithelioma papulosum cyprini* cells (EPC), Asian Grouper strain K (AGK) [31], and chinook salmon embryonic cells (CHSE) were all cultured in Leibovitz 15 (L-15) media, which was supplemented with 10% fetal bovine serum (FBS), L-glutamine, and gentamicin and maintained at 20 °C in L-15 medium (Invitrogen, Carlsbad, CA, USA) supplemented with 5% FBS, l-glutamine, and gentamicin. A recombinant IPN virus (rNVI-015) produced by reverse genetics was used. The virus was inoculated into 70–80% confluent CHSE cells followed by incubation at 15 °C and cultured until full cytopathic effects (CPE). The supernatant containing the virus was then harvested and clarified by centrifugation at 2500 rpm for 10 min. The concentration of the virus was estimated by titration in 96-well plates (Falcon, New York City, NY, USA). The obtained supernatant was used to infect CHSE cells to assess eIF2α phosphorylation (described below 2.3) as positive control.

### 2.2. Electroporation of Plasmids into EPC and AGK Cells

Eukaryotic expression plasmid pcDNA-wtcarpPKR expressing the wild-typecarp PKR and pcDNA-mutcarpPKR expressing a catalytically inactive PKR having a single mutation Lys419Arg (K419R) in the catalytic domain were kind gifts from Professor Gui [15]. For overexpression of carp PKR proteins, EPC cells were transfected by electroporation with 2 µg per 10^6^ cells of the wild type construct pcDNA-wtcarpPKR, the mutated form at the catalytic site pcDNA-mutcarpPKR or only the backbone plasmid pcDNA3.1-myc-His (Invitrogen, Carlsbad, CA, USA). Transfection was performed using the Neon transfection system (Invitrogen) with one pulse of 1200 V for 40 ms. After transfection, cells were kept at 20 °C for 3 days until further experiments. The three plasmids were designated wtPKR, mutPKR, and pcDNA3.1 corresponding to the pcDNA-wtcarpPKR, pcDNA-mutcarpPKR, and pcDNA3.1-myc-His, respectively.

### 2.3. Western Blot

Transfected cells were grown in 6-well plates and harvested for protein extraction. Cells were lysed using the CelLytic M reagent (Sigma-Aldrich, St. Louis, MO, USA) and scraped from the plates. Lysates were separated in 12% NuPAGE Bis-Tris gels (Invitrogen) and transferred to the PVDF membrane using Trans-Blot SD semi-dry transfer cell (BioRad, Hercules, CA, USA). Membranes were blocked for 2 h using 5% dry milk in TBST (0.02 M Tris-HCl, 0.9% NaCl, 0.05% Tween 20, pH 7.6). Polyclonal antibody against phosphorylated eIF2α (p-eIF2α) (Invitrogen), actin (Sigma) and mouse anti-c-myc monoclonal antibody was diluted in 2.5% dry milk in TBST and incubated overnight at 4 °C. Horseradish peroxidase (HRP) conjugated anti-rabbit or anti-mouse antibody (Cell Signaling, Danvers, MA, USA) diluted 1:2000 were added and incubated for 1 h. Final detection was achieved using the ECL Plus™ Western Blotting (WB) detection reagents and a Typhoon scanner (Amersham Biosciences, Little Chalfont, UK).

Quantification of eIF2α phosphorylation after transfection of pcDNA-wtPKR and pcDNA-mutPKR in EPC (2 experiments) and AGK cells (1 experiment) was done at 16, 24, and 40 h post transfection. The amount of p-eIF2α measured by densitometry (Typhoon Imager, GE Healthcare, Chicago, IL, USA) was quantified with ImageJ software, and the value was normalized against β-actin levels.

### 2.4. Apoptosis Assays

Annexin V-FLUOS (Sigma-Aldrich) in combination with PI staining was used to determine phosphatidylserine (PS) exposure in apoptotic cells using the Annexin V-FLUOS/PI Staining Kit (Sigma-Aldrich). Briefly, cells were washed with phosphate buffered saline (PBS), trypsinized, centrifuged and resuspended in labeling solution containing fluorescein-conjugated Annexin V and PI. Thereafter, they were incubated for 15 min in the dark at room temperature. This was followed by flow cytometry using Guava easyCyte™ Flow Cytometer (Merck Millipore, Burlington, MA, USA) and InCyte™ software version 0.2 (Merck Millipore). These studies were done in 2 independent experiments.

### 2.5. Measurement of Caspase-8 and -9 Activation

Measurement of caspase-8 and -9 activation was performed using Caspase 8/9 (active) FITC Staining Kit (Abcam, Cambridge, UK). Three days post plasmid transfection (dpt), EPC cells were incubated with FITC-IETD-FMK or FITC-LEHD-FMK, which irreversibly binds to activated caspase-8 or -9 in apoptotic cells. After incubation for 1 hour at room temperature, cells were washed twice with wash buffer and subsequently trypsinized, centrifuged, and resuspended in wash buffer and were subsequently subjected to quantification by flow cytometry using Guava easyCyte™ Flow Cytometer (Merck Millipore) and InCyte™ software version 0.2 (Merck Millipore). These measurements were carried out in 3 independent experiments.

### 2.6. Statistical Analysis

For the quantification of p-eIF2α and for the caspase assays, one-way analysis of variance was used to test for differences between transfected and non-transfected cells using GraphPad Prism 5.0 (GraphPad Software Inc., La Jolla, CA, USA).

## 3. Results

### 3.1. Carp PKR Overexpression Induce eIF2α Phosphorylation in EPC Cells

**Figure 1:**
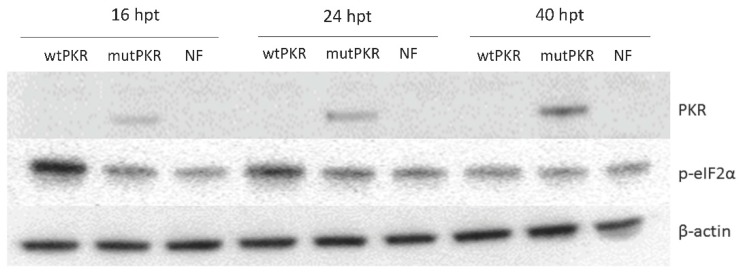
Expression of protein kinase R (PKR), phosphorylated eIF2α (p-eIF2α), and β-actin in *Epithelioma papulosum cyprini* (EPC) cells transfected with the wild type pcDNA-wtcarpPKR (wtPKR) and mutant pcDNA-mutcarpPKR (mutPKR), respectively. Negative control, non-transfected cells, were NF. Samples were analyzed at three time-points, 16, 24, and 40 h post transfection (hpt). PKR was not detected at any time point in wtPKR transfected cells, but positive in mutPKR cells, progressively increasing from 16 to 40 hpt. eIF2α was phosphorylated wtPKR cells, decreasing from 16 to 40 hpt. No difference in the phosphorylation levels of eIF2α between mutPKR and NF. β-actin was expressed at the same level from all samples at all sampling points.

**Figure 2:**
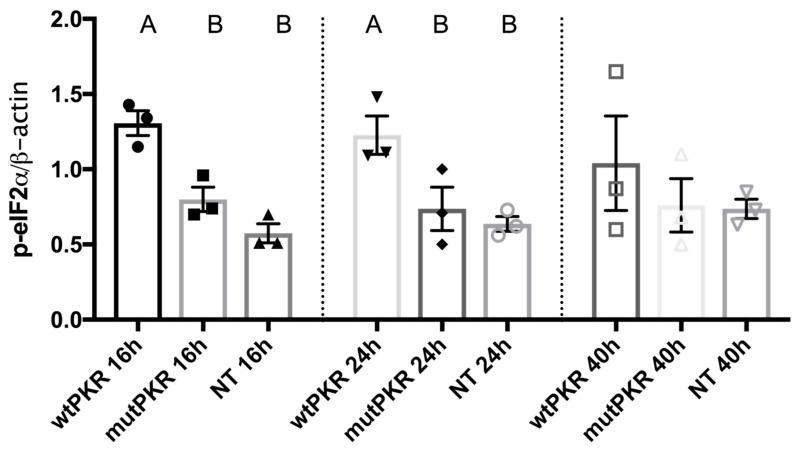
Quantification of eIF2α phosphorylation after transfection of pcDNA-wtPKR and pcDNA-mutPKR, and non-transfected controls in EPC and AGK cells at different time. p-eIF2α is measured by densitometry, expressed relative to β-actin. Representative data from three independent experiments are shown (mean ± SEM, *n* = 3). The different letters above the bars indicate significant differences (*p* < 0.05), and the different shapes on top of the columns indicate individual measurements. No differences were found at 40 h.

To determine the ability of PKR to phosphorylate eIF2α in piscine cells, we overexpressed the wild type wtPKR and the catalytically inactive mutant mutPKR in EPC cells. WB analysis showed that mutPKR was expressed as early as 16 hpt and expression levels continued to increase until 40 hpt. Conversely, PKR from the wtPKR transfected cells was not detectable by WB (Figure 1). Further wtPKR expression resulted in phosphorylation of eIF2α in EPC cells seen as strong phosphorylation of eIF2α at 16 hpt (Figure 1). Phosphorylation levels decreased at 24 hpt and dropped to similar levels as with negative control (non-transfected) at 40 hpt, considered as background levels. In contrast, mutPKR did not result in phosphorylation of eIF2α above background levels (Figure 1). β-actin was used to normalize the level of p-eIF2α phosphorylation for three independent experiments. A positive control (infection with infectious pancreatic necrosis virus) was included to demonstrate typical eIF2α phosphorylation as a result of virus infection (Supplementary Figure S1).

p-eIF2α was quantified using a Typhoon imager and expressed relative to β-actin (normalized) for each experiment. mutPKR was not different from non-transfected cells at any time point post transfection (Figure 2).

### 3.2. Carp PKR Overexpression Induce Apoptosis in EPC

**Figure 3:**
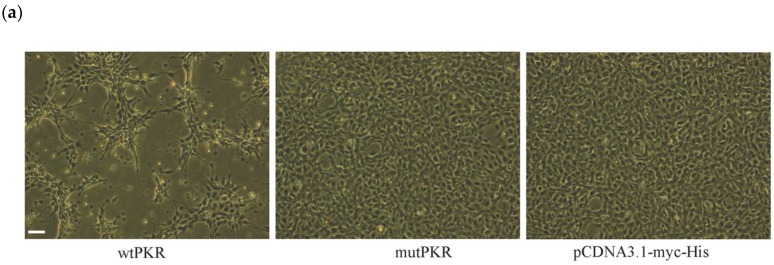

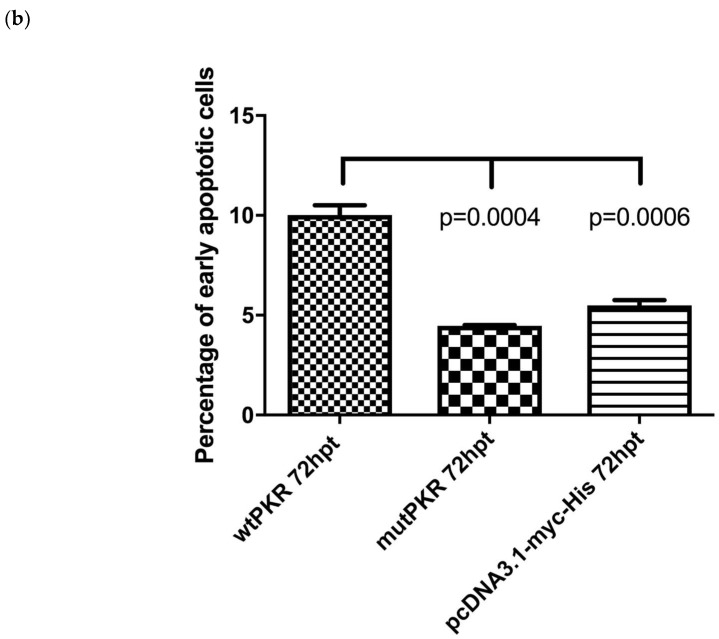
Cytopathic effects (CPE) and the percentage of apoptotic cells in EPC cells transfected with the wtPKR, mutPKR, and pcDNA3.1-myc-His plasmids (controls), respectively, 72 h post transfection. (**a**) Distinct CPE in cells transfected with the wtPKR. Cells transfected with mutPKR and pcDNA3.1-myc-His had insignificant CPE and both showing confluent monolayers, 72 h post transfection. Bar = 10 μm. (**b**) Flow cytometry analysis of Annexin V-Fluos and propidium iodide staining of apoptotic cells at 72 h post transfection. Each bar represents the average results of two independent experiments, three replicates in each. Percentage apoptotic cells in the wtPKR transfected cells was two-fold higher than in mutPKR cells (*p* = 0.0004) and compared to empty plasmid control, at 3 days post transfection (dpt). There was no significant difference between mutPKR and pcDNA3.1-myc-His transfected cells.

The cellular responses to overexpression of PKR in EPC cells were first assessed morphologically by comparing responses post transfection for wtPKR, mutPKR, and pcDNA3.1 (control) plasmids. There was distinct cell death in the wtPKR transfected cells at 72 h post transfection (hpt) (Figure 3a) while cells transfected with mutPKR did not show CPE and the cell monolayer was confluent and similar to cells transfected with the negative control (pcDNA3.1 plasmid), all observed at 3 dpt. To better understand the basis for the morphological changes observed, transfected cells were prepared for Annexin V/PI Staining followed by flow cytometry analysis, and we found that wtPKR transfected cells had about 10% apoptotic cells, annexinV-positive and PI-negative, in contrast to mutPKR and pcDNA3.1 controls that both had less than 5% apoptotic fluorescent cells (Figure 3b). Thus, overexpression of PKR results in induction of apoptosis while catalytically inactive PKR (mutPKR variant) did not result in cell death in EPC cells.

### 3.3. Caspas-8 and -9 Are Activated in PKR-Induced Apoptosis

**Figure 4:**
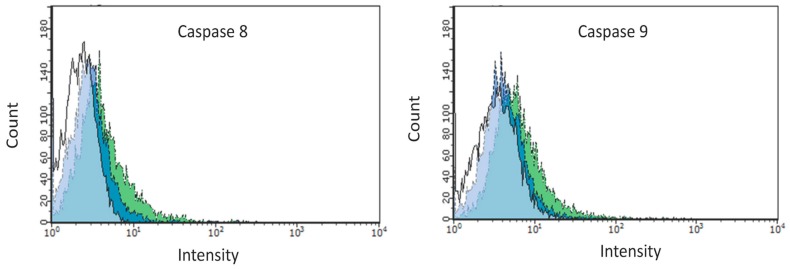
Activated caspase-8 and -9 positive cells were analyzed by flow cytometry at 3 days post transfection. Representative histogram showing the increase in caspase-8 and -9 staining in mutPKR (blue) and wtPKR (green) transfected cells relative to non-transfected control cells (white, in front).

**Figure 5:**
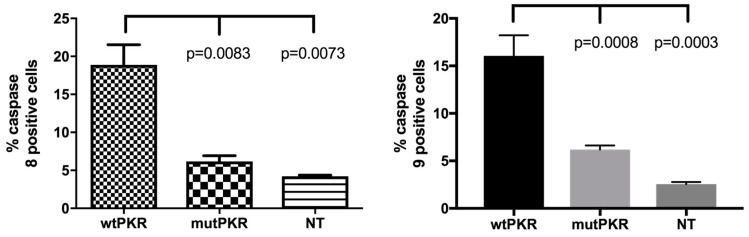
Percentage activated caspase-8 positive cells was about three-fold higher in the wtPKR transfected cells than in mutPKR cells (*p* = 0.0083), while activated caspase-9 was 2.5-fold higher in wtPKR compared to mutPKR transfected cells (*p* = 0.0008). The *p*-values for wtPKR versus non-transfected (NT) are also shown while p values for mutPKR versus NT were both >0.05.

On this basis, we went on to elucidate the underlying mechanisms of cell death, with a focus on receptor-mediated (extrinsic) or mitochondrial (intrinsic) pathway-induced apoptosis. With an aim to differentiate between the two pathways we measured activation of caspase-8 and -9 in EPC cells transfected with the different plasmids, and again cellular responses by flow cytometry. When incubated with FITC-IETD-FMK (caspase-8) or FITC-LEHD-FMK (caspase-9), we found increased fluorescence intensities for both caspase-8 and -9 in wtPKR transfected cells (Figure 4 and Figure 5). This is in contrast to the mutPKR transfected cells that had a fluorescence intensity level not different from the non-transfected cells (Figure 4). Quantitatively, we found 18.9% caspase-8 positive cells and 16.1% caspase-9 positive EPC cells in the wtPKR group. This was significantly higher than the non-transfected cells (Figure 5). Conversely there were 6.17% activated caspase-8 and 6.19% activated caspase-9 positive EPC cells in the mutPKR transfected cells that were not significantly different from the non-transfected control cells, indicating that mutational changes in the catalytically inactive mutPKR could have reduced the ability of PKR to activate caspase-8 and -9 (Figure 5).

## 4. Discussion

Here we show that overexpression of PKR in absence of a virus infection results in phosphorylation of eIF2α and induction of apoptosis that also involves activation of caspase-8 and -9. Overexpression of a catalytic domain mutated variant of PKR does not result in eIF2α phosphorylation and no induction of apoptosis.

Our findings are corroborated by several studies that have shown that PKR overexpression induces eIF2α phosphorylation in the absence of viral infection or introduction of exogenous dsRNA [15,32,33]. Koromilas et al. [33] showed that overexpression of PKR in NIH 3T3 mouse cells resulted in increased elF2a phosphorylation that led to increased cell death, while Chong et al. [34] showed that PKR overexpression led to increased eIF2α phosphorylation resulting in growth suppression of yeast cells. In fish cells, Liu et al. [15] showed that PKR overexpression in *Carassius auratus* blastula embryo cells (CAB) increased eIF2α phosphorylation in the absence of exogenous dsRNA but these authors did not study cell death of apoptosis induction.

PKR was not detected in the wtPKR transfected cells unlike the mutPKR transfected cells that efficiently expressed high PKR levels detected by WB. This is in line with Barber et al. who also observed that PKR was inefficiently expressed from wtPKR transfected green monkey COS-1 cells (*Cercopithecus aethiops*) compared to the mutPKR transfected cells that had 30- to 40-fold higher expression levels than wtPKR transfected cell. Protein stability measurements and primer extension analysis showed that PKR expression was autoregulated at mRNA translation level resulting in its inefficient expression in wtPKR transfected cells. They further observed that its regulation was highly affected by mutation either in the catalytic or N-terminal regulatory domains resulting in high expression levels in the catalytic inactive form but was inefficiently expressed in the wild type form due to mRNA translation autoregulation. Different scientists have reported similar autoregulation of PKR mRNA translation in different cell lines [34,35,36,37]. It is likely that the mutation from Lys_419_ to Arg_419_ introduced in the catalytic domain of the mutPKR variant used in this study increased the expression of PKR detected by WB, while the low expression levels detected in the wtPKR were due to autoregulation of mRNA translation of PKR. Furthermore, this suggests that the regulatory mechanisms involved in controlling PKR expression are conserved across the vertebrate taxa.

We have previously used the Annexin V/propidium iodide staining method to detect apoptosis after infectious pancreatic necrosis virus infection in fish cells [38]. However, unlike previous studies that link PKR-mediated apoptosis with viral infections [39,40] in mammalian cells, in the current study we show for the first time that PKR overexpression induces apoptosis without viral infection in fish cells.

Finally, it has been shown in mammalian cells that eIF2α phosphorylation by PKR leads to activation of caspase-8 that causes apoptosis via FADD [41] and caspase-9 that uses cytochrome-c and Apaf1 to induce cell death [18,42]. To our knowledge, there are no studies shown to link PKR induced apoptosis with the caspase-8 and -9 pathways in fish cells. To address this, we compared caspase-8 and -9 activation levels in the wtPKR transfected cells with mutPKR transfected cells. Our findings show that both caspase-8 and -9 were significantly activated in the wtPKR transfected cells, which corresponded with a highly significant increase in CPE. On the contrary, mutPKR transfected cells had no increase in the activation levels of caspase-8 and -9 being similar with the non-transfected control cells that had no CPE. Therefore, these findings suggest that apoptosis detected in the wtPKR transfected cells was induced by PKR via the caspase-8 and -9 pathways. In these studies, we did not include a chemical that induces apoptosis (like staurosporine [38]) and results should be viewed in this light. The high percentage of apoptotic cells and the contrasting findings relative to mutPKR and controls are in support of our view that the differences observed between wtPKR and the mutated forms clearly shows that overexpression of PKR induces apoptosis in the cell line tested and that the catalytically active domain plays a role in these processes. The fact that these studies were repeated with the same outcome are in favor of our interpretation.

In summary, these data suggest that PKR induced apoptosis via the caspase-8 and -9 pathways is a conserved cell death mechanism among vertebrates. Future studies should seek to consolidate these findings using gene-editing tools such as the CRISPR-Cas9 or TALEN technologies in order to underpin the functional roles of the individual genes involved in the apoptosis signaling pathway induced by PKR in fish cells.

## Supplementary Materials

The following are available online at http://www.mdpi.com/1999-4915/10/10/526/s1. Figure S1: eIF2α phosphorylation in CHSE-214 cells following IPN virus infection as positive control.
