# Supplementary material for: Apoptosis Induction by dsRNA-Dependent Protein Kinase R (PKR) in EPC Cells via Caspase 8 and 9 Pathways
Source: Viruses. 2018 Sep 27;10(10):526. doi: 10.3390/v10100526 (PMC6213184; doi:10.3390/v10100526)
Supplement: Supplementary file 1 [file viruses-10-00526-s001.pdf]

Supplementary Figure 1.

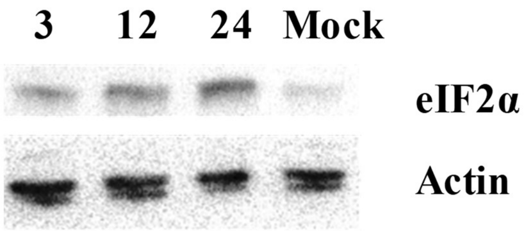

Supplementary Figure 1. eIF2α phosphorylation in CHSE-214 cells following IPN virus infection as positive control. The numbers represent time in hours post infection. eIF2α was induced already at 3 hpi increasing in up to 24 hpi. Western blot was performed using rabbit antibodies against the phosphorylated form of eIF2α. Equal amounts of protein were loaded in each lane (7μg total protein).
